# Bacterial extracellular vesicles target different bacterial species, impairing cell division and diminishing their pathogenicity

**DOI:** 10.1073/pnas.2416652122

**Published:** 2025-04-29

**Authors:** Yu Kawagishi, Kazunori Murase, Anna Grebenshchikova, Junpei Iibushi, Chang Ma, Teresia M. Kimeu, Atsuko Minowa-Nozawa, Takashi Nozawa, Ichiro Nakagawa

**Affiliations:** ^a^Department of Microbiology, Kyoto University Graduate School of Medicine, Kyoto University, Sakyo-ku, Kyoto 606-8501, Japan; ^b^Department of Bacteriology I, National Institute of Infectious Diseases, Tokyo 162-8640, Japan; ^c^Center for Health Security, Kyoto University Graduate School of Medicine, Kyoto University, Sakyo-ku, Kyoto 606-8501, Japan

**Keywords:** *Escherichia coli*, extracellular vesicles, group A Streptococcus, cell division, pathogenicity

## Abstract

Extracellular vesicles (EVs) produced by bacteria contain many bacterial-derived molecules, which play an important role in host interactions and as mediators of bacterial communication. However, the role of EVs in interspecies interactions and their physiological and ecological significance are not well understood. In this study, we found that *Escherichia coli* EVs inhibit the growth of group A *Streptococcus* (GAS; *Streptococcus pyogenes*) by inducing defective cell division via the following processes. *E. coli* EVs first attach to the cell surface of GAS. In EV-attached GAS cells, multiple septa and Z-rings form in close proximity, which clearly differs from the typical cell division process. This is due to inhibition of peptidoglycan (PG) remodeling in the process after septum formation, in which the next cell division is initiated without completion of peripheral PG synthesis. Therefore, cell division proceeds while inducing cell elongation and cell separation failure, leading to growth inhibition. Furthermore, EV alters the expression of approximately 10% of all genes encoded on the GAS genome, and the diverse functions of these gene sets, which include replication, division, and metabolism, suggest that EVs have a variety of biological effects on the targeted bacterial cells. Notably, *E. coli* EVs significantly decreased the expression of genes involved in representative GAS virulence, such as *slo*, *nga*, and *hasA*, and also markedly attenuated the pathogenicity of GAS in mice. Our findings provide insight into the competitive functions of EVs between different bacterial species, expanding current knowledge on EV-mediated interspecies interactions.

## Significance

Bacterial extracellular vesicles (EVs) play a crucial role in host–bacteria and bacteria–bacteria interactions as delivery platforms or mediators. In bacterial communication, EVs have a dual function, being both cooperative and competitive. Here, we show that *Escherichia coli* EVs adhere to the surface of *Streptococcus pyogenes* and inhibit peptidoglycan (PG) remodeling, resulting in cell division defects and eventually growth inhibition. The finding that EVs target different bacterial species and may play a role in interspecies competition is important. Notably, the expression of numerous genes was altered in EV-treated bacteria, especially the repression of virulence genes, thereby diminishing pathogenicity. This highlights the potential of EVs to exploit the functional characteristics of EVs in the development of new antimicrobial agents.

Most bacteria naturally release spherical lipid-bilayered extracellular vesicles (EVs) of 20 to 400 nm in size as part of their normal growth process, and the process of EV production is considered to be ubiquitous among bacteria (1). Bacterial EV formation and release are conducted in a constitutive or regulatory manner, and several mechanisms have been reported or proposed (2), including outer membrane blebbing in Gram-negative bacteria (3, 4), bubbling cell death in Gram-positive bacteria (5), and explosive cell lysis in both Gram-negative and Gram-positive bacteria (6). In addition, EV production has been reported to be induced by intracellular and extracellular environmental triggers (4). Regarding bacteria-derived EVs, their composition and enclosed components have been well studied in terms of pathogenicity and survival strategies. Numerous bacterial products, including proteins, nucleic acids, and virulence-related or signal molecules, have been identified as cargos that are loaded onto EVs (7). These sorting cargo molecules are maintained in an environment similar to that inside the cell, and therefore bacteria have the advantage of transporting the molecules in a stable manner (e.g., protected from degradation) without being exposed to the extracellular environment (8, 9). Furthermore, various studies have been conducted on the functional aspects of EVs, revealing that released EVs are involved in multiple biological processes including virulence, export of cellular metabolites, nutrition acquisition, horizontal gene transfer, biofilm formation, and cell-to-cell communication (7, 10, 11). EVs containing virulence factors are incorporated into host cells and play an important role in modulation of host immunity and virulence, contributing to bacterial infection strategies (12–14). In addition to these functional roles of EVs in host–bacteria interactions, the role and significance of EVs in bacterial interactions are becoming clearer.

Bacteria promote their own fitness and survival through coordinative or competitive interactions with each other in various environments (15). Representative cooperative behavior among bacteria is an intercellular communication process termed quorum sensing. This is a well-known system in which bacteria send and receive chemical signals (autoinducers) to each other to control gene expression and cellular functions, thereby modulating the production or release of biomolecules and metabolites in the population in response to external environmental factors, such as cell density(16). Bacteria also possess various competing systems to eliminate other bacterial species, including bacteriocins, antimicrobial peptides (17), and the type VI secretion system, a molecular device for injecting effectors into competitors (18). Thus, the interactions between bacteria through cooperative and competitive systems are diverse and are an important strategy for their own survival.

In addition to these modes of interaction, recent studies have reported that EVs contribute to bacterial interactions by delivering their enclosed proteins, genetic material, and signal molecules to bacterial cells. For example, EVs mediate the transfer of encapsulated DNA between bacteria, facilitating horizontal gene transfer for the acquisition of antibiotic resistance (19) and pathogenicity (20). It has been reported that *Pseudomonas aeruginosa* communicate and coordinate group actions through quorum sensing molecules such as quinolones or lactones loaded onto EVs (21). In iron-limited environments, EVs can function as a tool for the capture of extracellular heme and its transport, playing a mutualistic role between bacteria (22, 23). In contrast to the coordinated functional role of EVs within a bacterial population, it has been reported that EVs also have the ability to kill other bacteria, which is associated with the activity of EV-derived peptidoglycan (PG)-degrading enzyme (24–26). Thus, EVs serve an important physiological and ecological role as mediators in bacterial cooperative and competitive interactions, but are not fully understood.

In this study, to further investigate the physiological effects and significance of EVs among unrelated species, we examined various bacterial species and found that *Escherichia coli* EVs inhibited the growth of Gram-positive bacteria, with a particularly notable effect on *Streptococcus pyogenes* (group A *Streptococcus*, GAS). GAS is a human-specific pathogen that sometimes causes life-threatening diseases such as necrotizing fasciitis and streptococcal toxic shock syndrome (27). Elucidating this mechanism may offer important insights into the prevention and treatment of GAS infections, and therefore we used GAS as a model species in this study. The mechanism of growth inhibition by *E. coli* EVs in GAS was further investigated in detail, revealing a defective cell division process with aberrant morphology. In EV-treated GAS, the remodeling of PG during cell division was inhibited and new septa were formed without sufficient elongation and normal division, resulting in multiple septa and a cell division defect. In addition, the impacts of EVs were found at the transcriptional level, revealing genome-wide alterations in gene expression. In particular, EVs decreased the gene expression of major GAS virulence factors and their activities, and pathogenicity in mice was also remarkably attenuated. Our findings expand current knowledge on EV-mediated interspecies interactions and may reveal new bacterial competition systems.

## Results

### *E. coli* EVs Inhibit Growth of Various Gram-Positive Bacterial Species.

**Fig. 1.**
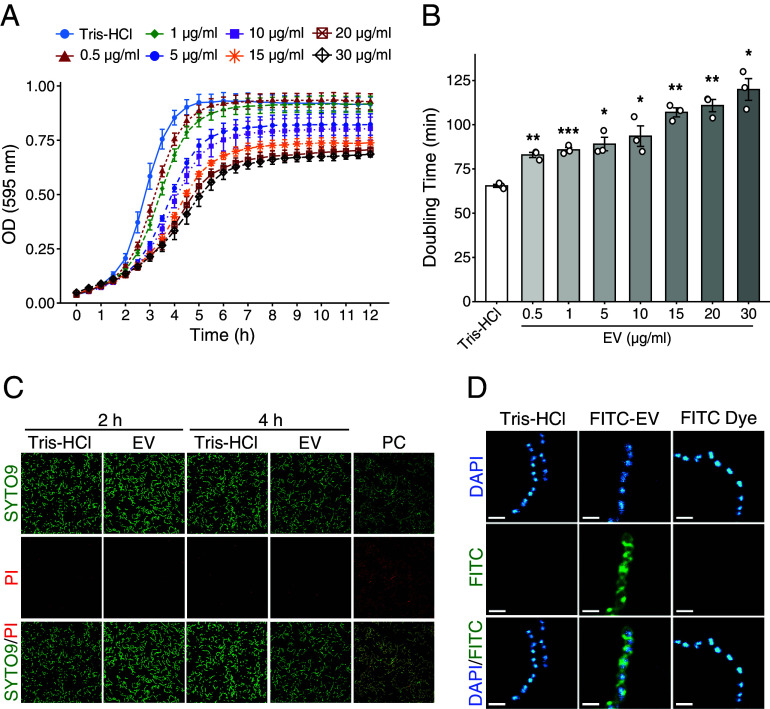
Growth inhibition of GAS by *E. coli* EVs. (*A* and *B*) Growth assay of strain JRS4 cells treated with different concentrations of EVs. The growth of strain JRS4 cells under each condition was measured at OD 595 nm, and the doubling times were estimated from OD values in the exponential part of each growth curve. The data represent the mean value of three biological replicates ± SEM. *P*-values were calculated by a two-tailed Student’s *t* test and denoted when the data were significant. *P < 0.05; **P < 0.01; ***P < 0.005. Treatment with Tris-HCl was used as a control. (*C*) Cell viability assay of EV-treated strain JRS4. JRS4 cells treated with EVs (10 µg/mL: 500 EV particles/cell) for 2 or 4 h were stained with PI/SYTO9 and observed by confocal microscopy. Green- or red-stained cells indicate total cells or dead cells, respectively. The estimated cell viability is shown in *SI Appendix*, Fig. S2. (*D*) EVs surrounding JRS4 cells. The cells were treated with FITC (green)-labeled EVs (10 µg/mL: 500 EV particles/cell) for 2 h at 30 °C and stained with DAPI (cyan). The cells were observed by confocal microscopy. (Scale bar, 1 µm.)

To investigate the biological activity of EVs derived from *E. coli* on other bacterial species, we first examined the effect of *E. coli* EVs on the growth of several bacterial species (*SI Appendix*, Fig. S1). Growth assays in the presence of *E. coli* EVs showed significant growth inhibition of all Gram-positive bacteria examined (*S. pyogenes*, *Streptococcus pneumoniae*, *Staphylococcus epidermidis*, and *Bacillus subtilis*), but no effect on the growth of *E. coli* itself. In particular, growth inhibition of *S. pyogenes* was more pronounced than for other bacterial species. EV-treated GAS JRS4 grew during the incubation time, but its growth efficiency was clearly lower at log phase than that of EV-untreated GAS (Fig. 1*A*), and growth inhibition was dose-dependent. The significant increase in cell doubling time during log phase also reflects the obvious delay in growth due to EV treatment (Fig. 1*B*), with the doubling time increasing by 54.5 min in 30 µg EV-treated cells. Although there was a clear decrease in growth in EV-treated GAS (approximately threefold lower OD values), the growth proceeded slowly and reached stationary phase even at high EV concentration. These results suggest that EVs may act bacteriostatically on GAS and suppress their growth efficiency. We next performed a Live/Dead assay to estimate the viability of JRS4 cells following treatment with EVs (Fig. 1*C* and *SI Appendix*, Fig. S2). After 2 or 4 h of treatment, although there was a notable difference in growth between EV-treated and untreated cells, neither stained with PI and there was no significant difference in the proportion of dead cells to total cells (approximately 2%). Furthermore, FITC-labeled EVs were observed uniformly covering the entire surface of JRS4 cells after 2 h of treatment (Fig. 1*D*). These results indicate that *E. coli* EVs interact with GAS without causing damage, and that EV itself or EV-derived cargo molecules may be involved in growth inhibition.

### *E. coli* EVs Induce Defective Cell Division with Multiple Septa in GAS.

**Fig. 2.**
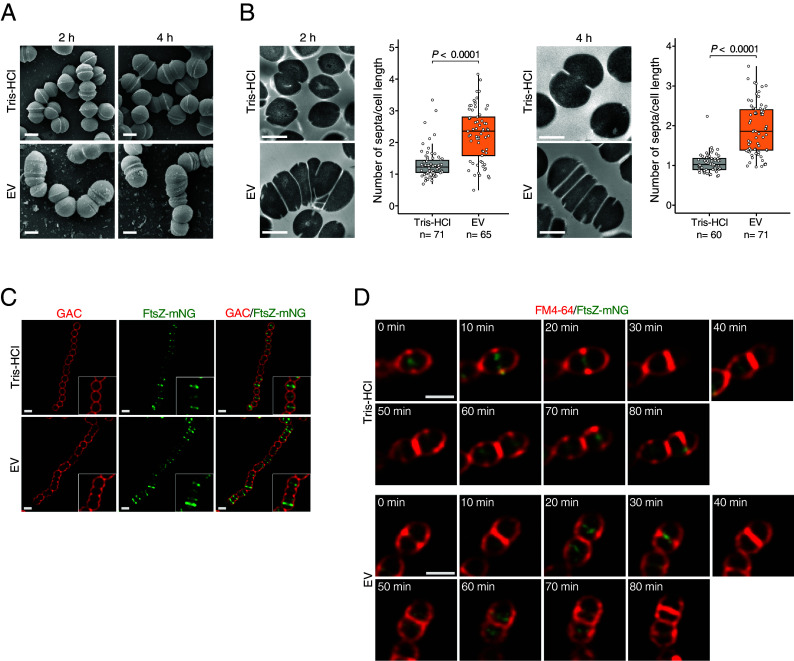
Defective cell division with morphological abnormalities in EV-treated GAS. The morphology of GAS cells treated with EVs (10 µg/mL: 500 EV particles/cell) for 2 or 4 h was observed using scanning electron microscopy (*A*) and transmission electron microscopy (*B*). The distribution of the number of septa per cell length was determined using ImageJ software, and the ratios are shown in box-and-whisker plots with individual data points (n = 56 to 66). Data are from three independent biological experiments. *P*-values were calculated by a two-tailed Student’s *t* test. (Scale bar, 500 nm.) (*C*) Localization of FtsZ in EV-treated GAS. JRS4 strain expressing FtsZ-mNG (green) was treated with EV (10 µg/mL: 500 EV particles/cell) or Tris-HCl for 2 h. Bacterial cell membranes were subsequently stained with anti-GAC antibody (red), and the cells were observed using confocal microscopy. The inset presents a magnified view of a representative area in the main image. (Scale bar, 1 µm.) (*D*) Time-lapse microscopy analysis of Z-ring formation in the cell division process. EV-treated JRS4 strain expressing FtsZ-mNG was stained with FM4-64 (red) and observed for the Z-ring formation process during cell division every 10 min.

Accurate DNA replication and cell division are necessary for normal bacterial growth. If these functions or processes are impaired, defective cell division occurs and growth is inhibited. To investigate whether the EV-induced inhibition of GAS growth may be due to defective cell division, we first analyzed cell morphology using scanning electron microscopy (SEM) and transmission electron microscopy (TEM) to examine whether EV-treated GAS cells display morphological changes. Morphological observations by SEM revealed that EV-untreated cells had a chain structure with uniform-sized spheres, whereas cells treated with EVs for 2 h and 4 h contained several cells that were partially expanded and showed heterogeneous spherical structures (Fig. 2*A*). TEM observations also revealed that the asymmetric heterogeneous spherical structures of GAS cells following EV treatment were due to the multiple septa formed in a single cell (Fig. 2*B*). The number of septa per cell was significantly higher in EV-treated cells (mean ratio: 2.26) compared with untreated cells (mean ratio: 1.32). Most EV-treated cells still possessed multiple septa after 4 h (mean ratio: 1.92), but these were decreased compared with the number of septa after 2 h. This is likely the result of growth of cells that were not affected by the EV-induced cell division disorder. These data suggested that EVs cause defective cell division with multiple septa formation in GAS, leading to growth inhibition. Bacterial cell division is generally initiated by the formation at midcells in a ring-like structure called the “Z-ring,” which is formed by polymerization of the tubulin homolog FtsZ (28). The Z-ring at the midcell organizes components necessary for cell division, and the septum forms at this site. To explore the mechanisms underlying EV-induced defective cell division, we used GAS expressing the ftsZ-mNeonGreen fusion protein (FtsZ-mNG) to investigate whether EV treatment affects the localization of FtsZ or Z-ring formation (Fig. 2*C*). Unlike untreated cells that localized FtsZ-mNG at midcells, EV-treated cells exhibited multiple Z-ring formations and multiple septum formations in close proximity along the long axis. We also performed time-lapse analysis of GAS cells to examine the formation process of the Z-ring over time following EV treatment (Fig. 2*D*). In EV-untreated cells, the Z-ring located at the midcell (0 min), after which the cell began elongation and septal synthesis (10 to 20 min). Once septum synthesis was complete, the cell constricted (30 to 40 min) and proceeded to the next cell division process (50 to 80 min). By contrast, EV-treated cells showed less constriction, resulting in a slight defect in cell separation (30 to 50 min). In the subsequent step, the cells formed new septum without sufficient elongation and separation (60 to 70 min), which led to aberrant cell morphology with multiple septa (80 min). These results suggest that EVs interfere with cell elongation and separation in GAS, resulting in cell division defects with multiple septa in close proximity.

### *E. coli* EVs Inhibit PG Remodeling.

**Fig. 3.**
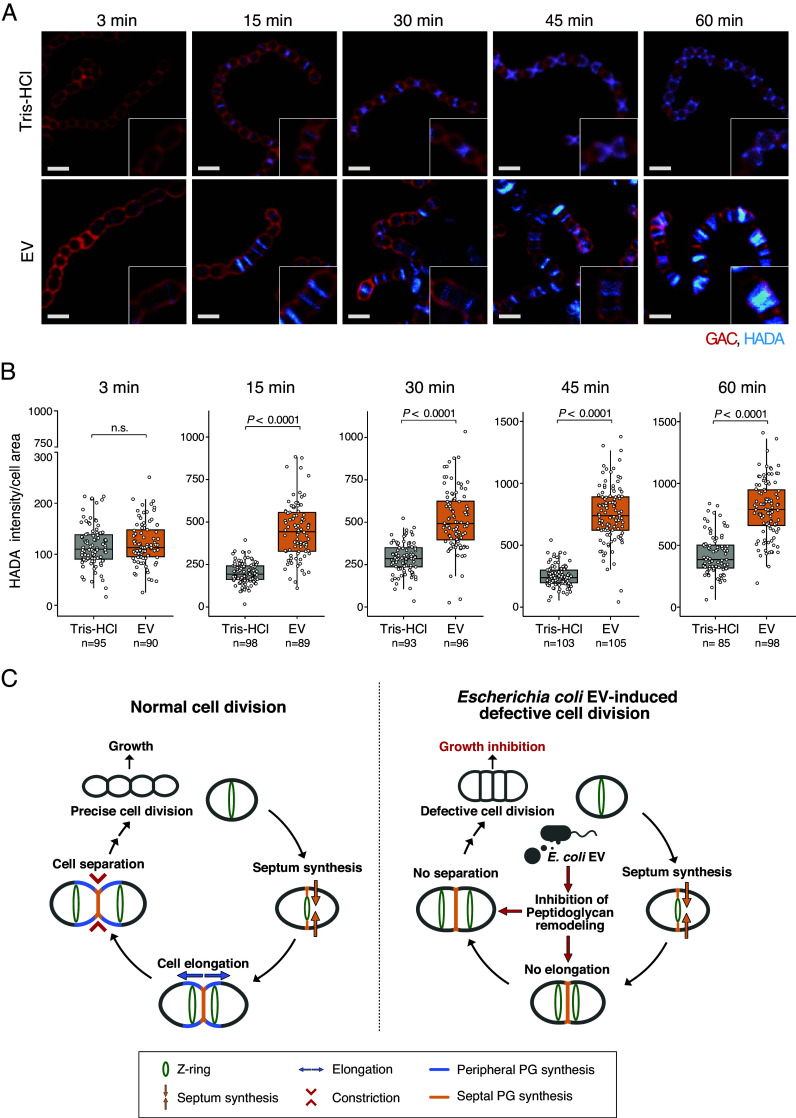
Inhibition of peptidoglycan (PG) remodeling by EV leading to a defective cell division process. Visualization (*A*) and quantification (*B*) of newly synthesized PG using fluorescent D-amino acid HADA in EV-treated GAS. After 2 h of EV treatment (10 µg/mL: 500 EV particles/cell), cells were labeled with 500 µM HADA (cyan) for 3, 15, 30, 45, or 60 min, and observed using confocal microscopy. Bacterial cell membranes were stained with anti-GAC antibody. The inset presents a magnified view of a representative area in the main image. (Scale bar, 1 µm.) The distribution of HADA intensity per single cell chain was measured using the MicrobeJ plugin, and the ratios are shown in box-and-whisker plots with individual data points (n = 45 to 92). Data are from three independent biological experiments. *P*-values were calculated by a two-tailed Student’s *t* test. (*C*) Hypothesized schematic representation of the EV-induced defective cell division process in GAS. During normal cell division, the Z-ring (green) is located at the midcell and the septum PG (orange) forms in the center at the initiation of cell division. Simultaneously, peripheral PG (light blue) is synthesized to form a hemisphere and elongate cells. Finally, the cells constrict and form a chain-like structure (*Left* panel). In EV-treated cells, PG remodeling does not function properly due to diminished hydrolase activity, and multiple septa in close proximity are formed as the cells attempt to proceed to the next step in the cell division process without sufficient cell elongation. Finally, the cells could not separate normally, leading to growth inhibition (*Right* panel).

PG is a major component of bacterial cell walls and plays a crucial role in cell shape maintenance, cell division, and cell elongation (29). To further investigate the defective cell elongation and separation in EV-treated GAS, we examined the effect of EVs on PG synthesis by labeling newly synthesized PG with the d-amino acid fluorescent probe HADA (Fig. 3 *A*, *B*). In EV-untreated cells, HADA was initially incorporated at the midcell during septal PG synthesis (3 to 15 min), and then expanded to the periphery during peripheral PG synthesis (30 to 45 min), and finally formed a new hemisphere in each daughter cell and was incorporated at the midcell during new septal PG synthesis (60 min). By contrast, in EV-treated cells, HADA was initially incorporated into the midcell (3 to 15 min), but accumulated at the midcell without spreading to the periphery (30 to 60 min). Furthermore, HADA in EV-treated cells accumulated in the septum at 15 min (2.23-fold), 30 min (1.77-fold), 45 min (3.01-fold), and 60 min (1.87-fold) compared with EV-untreated cells. These data suggest that septal PG synthesis still occurred in EV-treated cells, but not for peripheral PG, resulting in shorter distances between septa. These changes in PG dynamics indicate that EVs inhibit PG remodeling.

PG remodeling, the well-orchestrated coordination process of breaking down old PG and synthesizing new PG, is essential for cell elongation and daughter cell separation. To assess the impact of EVs on the balanced synthesis and degradation of PG, we first examined the growth of GAS by ampicillin-induced cell lysis assay. In GAS cells exposed to ampicillin, PG synthesis was inhibited, while PG degradation proceeded, leading to cell lysis and subsequent growth arrest. However, EV-treated cells maintained the growth even at high concentrations of ampicillin, requiring 1,000-fold higher concentrations than EV-untreated cells to completely inhibit the growth (*SI Appendix*, Fig. S3). This suggests that EV treatment reduced the PG hydrolase activity in GAS, preventing an imbalance between PG synthesis and degradation. We further performed PG zymography to evaluate the activity of PG hydrolase in GAS, and a transparent band indicating PG hydrolase activity was clearly attenuated in EV-treated cells compared to EV-untreated cells (*SI Appendix*, Fig. S4). These results suggest that EVs are associated with a decrease in PG hydrolase activity rather than interfering with the PG synthesis, thereby inhibiting PG remodeling. Taken together, PG remodeling does not function properly in EV-treated GAS cells due to diminished hydrolase activity, and multiple septa in close proximity are formed as the cells attempt to proceed to the next cell division process without sufficient cell elongation. Finally, it is inferred that the cells could not be separated normally, leading to growth inhibition (Fig. 3*C*).

### *E. coli* EVs Widely Alter the Expression of Genes in the GAS Genome.

**Fig. 4.**
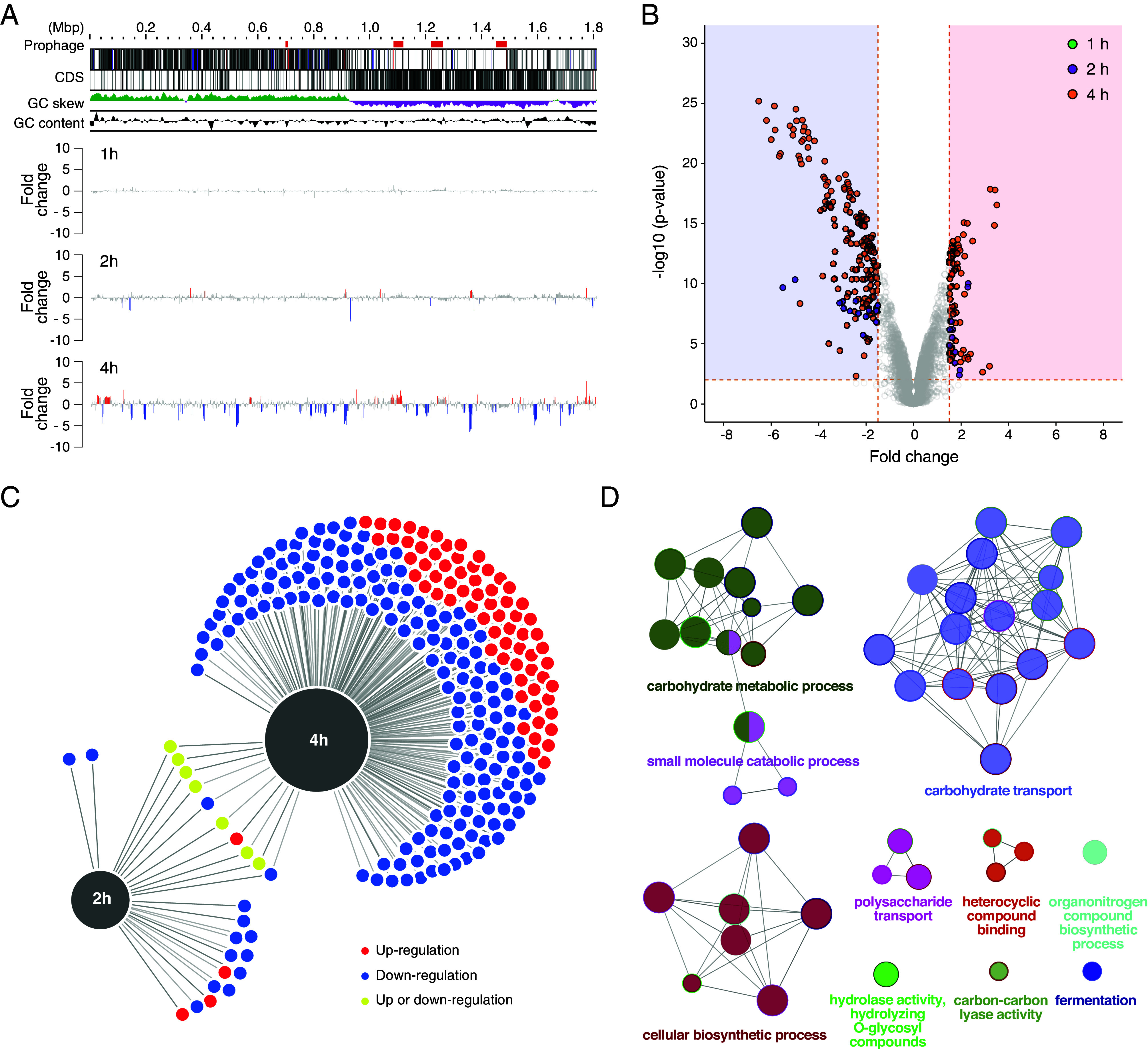
Genome-wide transcriptional profile of GAS treated with *E. coli* EVs. Genome coordinate map (*A*) and a volcano plot (*B*) depicting genes that are significantly upregulated (log2 FC > 1.5, *P*-value < 0.01) or downregulated (log2 FC < −1.5, *P*-value < 0.01) in EV-treated GAS relative to untreated cells. Plots in the red and blue shaded areas of the volcano plot indicate genes that were significantly upregulated or downregulated in EV-treated GAS, respectively. The plot colors indicate the data at each time point (1 h, green; 2 h, purple; 4 h, orange). (*C*) A force-directed graph comparing the variation pattern of DEGs obtained from RNA-seq data at each time point. The graph was generated by DiVenn tool v2.0. Blue and red nodes denote downregulated and upregulated genes between different time points, respectively. Yellow nodes denote upregulation in one time point but downregulation in another. (*D*) GO functional enrichment analysis of DEGs showing the downregulation in EV-treated GAS. The 170 downregulated genes in EV-treated GAS at 4 h were used for GO enrichment analysis (*SI Appendix*, Table S2), and the enriched GO terms and the pathway were visualized using the Cytoscape plug-in ClueGO with the following parameters: two-sided hypergeometric test, *P*-value cutoff = 0.05 and corrected by Bonferroni step down, min GO level = 3, max GO level = 8, GO grouping = true, and kappa score threshold = 0.4. The enrichment shows only significant pathways (*P*-value ≤ 0.05), and the values of *P* ≤ 0.05 indicate the node size. The node color code indicates the specific functional class, and the color represents various molecular pathways involved in the enrichment analysis of the analyzed DEGs. The most critical functional pathways defined in each group are shown.

The finding that EVs cause cell division defects implies a significant impact on the biological activity of target bacteria at the transcriptional level. Therefore, we performed RNA-seq analysis of EV-treated or untreated GAS, and identified the differentially expressed genes (DEGs; log_2_ fold-change (FC) > 1.5, log_2_ FC < −1.5, *P*-value < 0.01) at each time point. DEGs were detected at each time point and distributed throughout the entire genome rather than at specific loci (Fig. 4*A*). Furthermore, the number of DEGs was increased from 26 genes (11 activated, 15 repressed) at 2 h to 246 genes (72 activated, 174 repressed) at 4 h, which were found throughout the GAS genome (Fig. 4*B* and *SI Appendix*, Table S1). The repertoire of genes with significant expression variation differed slightly at 2, and 4 h, and only some gene expression patterns showed linkage over time (Fig. 4*C*). At 4 h, EVs affected the gene expression of almost 10% of the GAS genome, with most of these genes being suppressed. In addition, GO enrichment analysis highlighted the repressive effects on multiple biological processes and molecular functions, particularly carbohydrate metabolism/transport, enzymatic activity (e.g., hydrolase activity), and toxin activity, which are triggered at the transcriptional level in the EV-treated GAS (Fig. 4*D* and *SI Appendix*, Table S2). These dramatic changes in gene expression profiles suggest that EVs may directly or indirectly interfere with a broad range of endogenous biological activities besides impairing the cell division process.

Notably, major virulence-related genes were also suppressed in EV-treated GAS, e.g., hyaluronic acid capsule synthesis (*hasABC*), the hemolytic exotoxin streptolysin O (*slo*), NAD glycohydrolase (*nga*), streptolysin S precursor (*sagA*), endoglycosidase (*endoS*), and streptokinase A (*ska*). These gene expression levels are highly associated with invasiveness and anti-phagocytosis, which contribute to deep colonization and resistance to neutrophil killing in GAS (30–33). Individual transcriptional profiles, reconfirmed by qPCR, revealed that the expression levels of these genes were significantly reduced by approximately 2.5-fold or more in EV-treated GAS (*SI Appendix*, Fig. S5), suggesting that EVs drastically suppress the expression of genes critical for pathogenicity, and may lead to the attenuation of GAS virulence.

### *E. coli* EVs Reduced the Virulence Activities of GAS and Attenuated Its Pathogenicity in Mice.

**Fig. 5.**
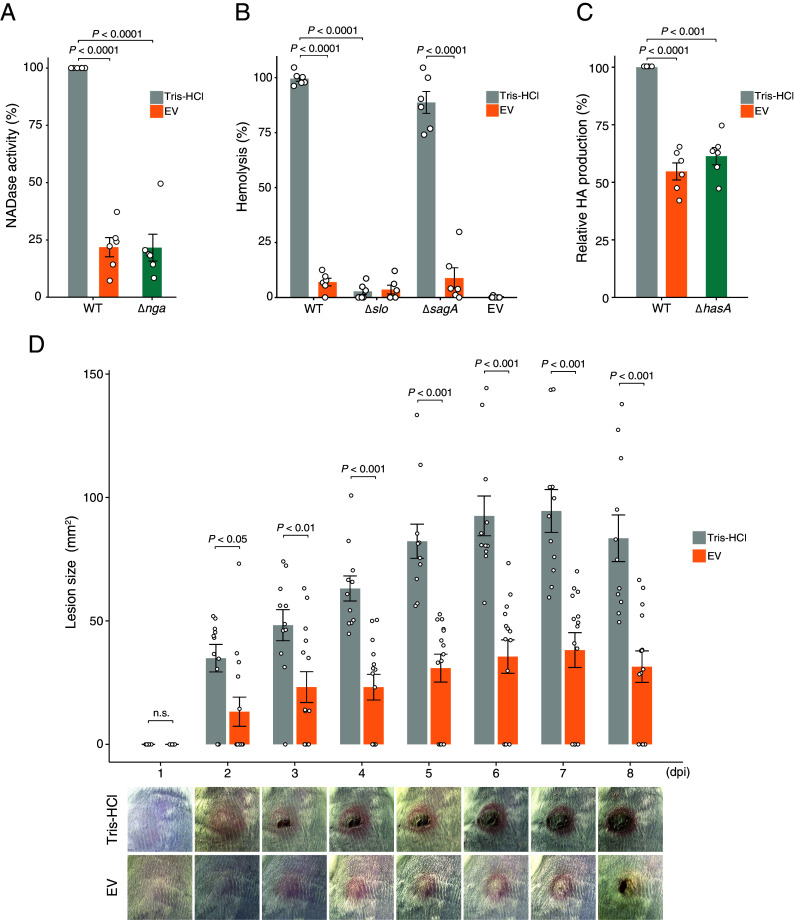
In vitro and in vivo assessment of the pathogenicity of EV-treated GAS. The activities of virulence factors in JRS4 were evaluated by an NADase assay (*A*), a hemolysis assay (*B*), and HA capsule production (*C*). Detailed methods for each assay are described in the Materials and Methods. NADase activity and HA production were calculated relative to the WT strain treated with Tris-HCl. In the hemolytic assay, the percentage of hemolysis in each sample was calculated by setting the positive control, which completely lysed the RBCs, at 100%. Analyzed data are shown in the bar plots with individual data plots. The deletion mutants of genes involved in each virulence activity were used in the assay (*hasA* gene mutant for HA production, *nga* gene mutant for NADase assay, and *slo* or *sagA* gene mutant for hemolysis assay). (*D*) Assessment of GAS pathogenicity in the mouse skin infection model. ICR mice were injected subcutaneously with invasive GAS strain SSI-1 treated with EV (n = 14) or Tris-HCl (n = 11). The lesion area around the injection site was observed daily for 8 d, and the representative images are shown (see *SI Appendix*, Fig. S7 for images of all mice at 8 dpi). In the mice infected with Tris-HCl-treated GAS, the skin redness indicates inflammation due to the infection, and lesions formed with each passing day. The lesion size was quantified using ImageJ software, and the quantified data are shown in bar plots with individual data plots. All data were obtained from the analysis with at least two biological replicates ± SEM. *P*-values were calculated by a two-tailed Student’s *t* test.

We next evaluated the activity of streptolysin S (SLS), streptolysin O (SLO), and NAD-glycohydrolase (Nga), the major virulence factors of GAS that showed differential gene expression following EV treatment. In the NADase assay, the NADase activity of EV-treated cells was approximately fourfold lower than that of untreated cells (Fig. 5*A*), reflecting the differential expression of *nga* at the transcriptional level. Similarly in the hemolysis assay, EV-treated cells showed a significant decrease in hemolytic activity compared with untreated cells (Fig. 5*B*). At the transcriptional level, both the *slo* and *sagA* genes involved in hemolytic activity were downregulated following EV treatment. However, the *sagA* mutant exhibited significant hemolysis, indicating that the hemolytic activity in this assay is SLO-dependent. Hyaluronic acid (HA) production was also significantly reduced in EV-treated cells, similar to the *hasA* gene deletion mutant (Fig. 5*C*), indicating the EV-induced decrease in capsule formation ability. These data indicate that EVs suppress the production of various molecules associated with the pathogenicity of GAS.

To evaluate the impact of EVs on the virulence of GAS in vivo, we used invasive strain SSI-1, which is known for its high capsule production and high virulence in mice (34). EVs also inhibited the growth of strain SSI-1, although to a lesser degree than strain JRS4 (*SI Appendix*, Fig. S1). In addition, HA production, NADase activity, and the hemolytic activity of SSI-1 were also significantly decreased by EV treatment (*SI Appendix*, Fig. S6 *A*–*C*). In the mouse skin infection model, ICR mice were subcutaneously injected with SSI-1 treated with EVs or Tris-HCl, and we observed and quantified the lesion area with abscess formation (Fig. 5*D*). In all mice infected with Tris-HCl-treated GAS (11/11 mice), lesions developed on their backs at 2 d, and progressed to larger lesions averaging 83.5 mm^2^ with obvious abscesses at 8 d. By contrast, although more than half of the mice infected with EV-treated GAS (10/14 mice) developed red lesions within 2 d, they did not progress to larger lesions, averaging 31.5 mm^2^ at 8 d. The remaining four mice showed no symptoms. Mice infected with EV-treated GAS exhibited significantly smaller lesions from day 2 onward compared with mice infected with Tris-HCl-treated GAS. A significant difference in the mean lesion size was also evident among mice with lesions (*SI Appendix*, Fig. S7*B*), indicating that lesion formation and progression were remarkably suppressed in mice infected with EV-treated GAS (*SI Appendix*, Fig. S7*A*).

## Discussion

Bacterial EVs have a wide variety of functional roles, and contain a diverse array of molecules, including proteins, nucleic acids, and signaling molecules (7). In particular, in host–bacteria interactions, EVs play a role in infection by transporting the encapsulated virulence factors to the host cell (12). EVs have also been reported as a tool for communication between bacteria to exchange cargo molecules, and are considered to be one of the important factors in establishing bacterial interactions within a population (35).

A key finding of this study was the demonstration that EVs can induce growth inhibition with unusual cell division against target bacteria. Bacterial growth requires precise DNA replication and cell division, which are strictly regulated both temporally and spatially. The surfaces of GAS and other Gram-positive bacteria are densely covered with PG, and PG synthesis and remodeling are essential for each step of the cell division process, namely septum formation, elongation, and segregation (36). Investigation into the cell division process in this study revealed that *E. coli* EV-treated GAS cell division proceeds without sufficient elongation and normal segregation, forming morphological abnormalities with multiple septa, as shown by microscopy observations. Similar division defects and cell morphology changes have been reported in streptococci with defects in PG-degrading enzymes (37, 38), the serine/threonine protein kinase signaling system (37, 39, 40), and DivIVA protein (41, 42), which regulate cell elongation and segregation. In these reports, it is noteworthy that depletion, reduced expression, or defective localization of PG-degrading enzymes is the cause of cell division defects. PG-degrading enzymes are required for PG remodeling, and their deficiency leads to impaired growth and cell division (29). Our analysis also showed that EV-treated GAS displayed decreased activity or production of PG hydrolase (*SI Appendix*, Fig. S4) and altered PG dynamics during cell division (Fig. 3*B*). Although low-dose flavomycin treatment, which inhibits a specific PG synthesis pathway, has also been reported to induce multiple septa in GAS cells (43), our analysis showed that such multiple septa formation was significantly more frequent in EV-treated cells. In contrast, flavomycin treatment primarily led to abnormal FtsZ dispersion or the absence of Z-ring formation (*SI Appendix*, Fig. S8). These findings indicate that both EV and flavomycin treatments lead to FtsZ mislocalization, but the mechanisms underlying PG remodeling inhibition likely differ. While the potential impact of EV treatment on PG synthesis remains to be explored, our data suggest that the primary effect of EVs on PG is due to a decrease in PG degradation activity rather than inhibition of PG synthesis. Particular hydrolase enzymes are required for PG remodeling, and their deficiency leads to growth and cell division defects (37, 44). Hence, we expect that the effect of EVs on PG-degrading enzymes leads to inhibition of PG remodeling, which is a key factor leading to cell division defects and the associated growth inhibition of GAS.

Another important finding was that EVs dramatically reduced pathogenicity while inhibiting the cell division of target bacteria. RNA-seq analysis revealed that EVs caused gene expression variation across approximately 10% of the GAS genome and suppressed many genes involved in diverse biological processes, including metabolism, replication, and virulence (Fig. 4 and *SI Appendix*, Table S1). It is well known that *covRS*, one of the two-component regulatory systems in GAS, regulates the gene expression of approximately 15% of the GAS genome (45), and the DEGs found in this study included many genes under its control, such as *hasA*, *slo*, *nga*, *sagA*, *endoS*, and *ska*. However, because there was no change in gene expression of *covRS* in EV-treated GAS, the genome-wide gene expression variation caused by EVs may be mediated by another transcriptional regulatory factor or epigenetic changes. Alternatively, this dramatic variation was similar to responses to antibiotic stress (46), oxidative stress, and heat stress (47), suggesting that EVs may act as stimuli or stresses on GAS, causing large-scale gene expression variation.

Notably, the expression of major virulence-related genes was suppressed in EV-treated GAS, and their production and activity were also decreased. As shown in Fig. 5, *E. coli* EVs contribute to the diminished pathogenicity of invasive GAS strain SSI-1 in mice. SSI-1 is known to produce high levels of hyaluronan capsule and toxin and to cause fatal infections in mice even at low bacterial cell counts (34), but EVs effectively suppressed SSI-1 growth and hyaluronan capsule production and greatly reduced lesion formation in a mouse skin infection model. In this skin infection model, it has been reported that the HA capsule is an important virulence factor for escaping neutrophil clearance and contributes significantly to colonization and lesion formation (31). Therefore, the decrease in lesion formation in this study may be attributed to reduced HA capsule and growth efficiency, resulting in unsuccessful colonization in the early stages of acute infection.

Previous studies have reported that the cargo of EVs, such as PG-degrading enzymes, exhibit bactericidal activity against other bacterial species (24–26), indicating the potential of EVs as antimicrobial agents. In the treatment of meningitis and sepsis caused by bacterial infection, it is necessary to reduce mortality and minimize the effects on the nervous system by inhibiting the release of bacterial components and toxins (48). The bacteriostatic effect of *E. coli* EVs, a key finding in this study, may contribute to the inhibition of the release of various bacterial-derived molecules that induce such inflammation. Furthermore, the effects of EVs on target bacteria demonstrated in our study go beyond simple growth inhibition, interfering with multiple critical functions of bacteria, including cell division, metabolism, and virulence. Further investigation on the detailed mechanisms and factors responsible for these effects are expected to facilitate the development of new antimicrobial therapies against bacterial infections through the effective use of EVs.

In EV-mediated bacterial communication, studies have reported that EVs transport cargo molecules such as quorum sensing signals (49) and plasmid DNA (50) via adhesion to target cells in *P. aeruginosa* and *E. coli*, and that these molecules play functional roles in promoting coordinated behavior and drug resistance. However, the surface structures of *E. coli* EVs and GAS cells with dense PG analyzed in this study are clearly different. Therefore, we infer that EV association may trigger a dynamic change in the biological process toward the target cells, rather than the cell division defects and diminished pathogenicity found in this study being due to the effect of the cargo molecules from EVs. This implies that EVs may promote competitive relationships among bacteria and contribute to dynamic changes in bacterial community structure in environments containing different bacterial species, such as the human microbiome. Although our understanding of the EV-mediated competition among different bacteria is still in its infancy, the findings in this study enhance our current knowledge of the physiologically and ecologically important roles of bacterial EVs in bacterial population networks in natural environments.

## Materials and Methods

### Bacterial Strains and the Culture Conditions.

The bacterial strains and plasmids used in this study are listed in Supplementary *SI Appendix*, Table S3. Additionally, the methods used to construct the mutant strains in this study are detailed in *SI Appendix*. For cultivation of GAS, bacterial cells were routinely grown at 37 °C in Todd–Hewitt broth (BD Bacto) supplemented with 0.2% yeast extract (THY) or THY agar plates; both were supplemented with 100 µg/mL spectinomycin (Spec) or 1 µg/mL erythromycin (Em) when necessary. The culture conditions for each assay are described below where appropriate.

### Preparation of EVs from *E. coli*.

EVs were isolated as described previously (51). Briefly, *E. coli* strain MG1655_GST-HlyF_ was grown in Luria–Bertani (LB) broth for 24 h at 37 °C. The culture supernatant was then obtained by centrifugation for 15 min at 7,500 g at 4 °C, and the supernatant was then filtered through a 0.45 μm pore membrane (Millipore) to obtain cell-free supernatants. The filtered supernatant was further ultracentrifuged for 2 h at 150,000×*g* at 4 °C, and the pellets were washed three times with phosphate-buffered saline (PBS). The isolated EVs were further purified using the OptiPrep (Sigma-Aldrich) density gradient method (see *SI Appendix* for details), and the purified EVs were then used for subsequent experiments. The protein concentration of EVs was determined using a BCA Protein Assay Kit (Nacalai Tesque) according to the manufacturer’s instructions. In addition, we also performed the nanoparticle tracking analysis using the NanoSight NS300 (Malvern, UK) and estimated the number of particles from the protein concentration.

The number of EV particles at the 10 µg/mL protein concentration primarily used in this study corresponds to approximately 4×10^10^ particles. Approximately 500 particles/cell were therefore used in the experiments involving EV treatment.

### Growth Assay.

For growth assessment of GAS with or without treatment with EVs, the bacterial cells grown to an OD 600 nm of 0.1 were treated with each concentration of EV (0.5, 1, 5, 10, 15, 20, and 30 µg/mL) and further incubated at 37 °C in THY broth for 12 h without shaking. Growth analysis was performed on the VICTOR Nivo Multimode Microplate Reader (PerkinElmer) at 37Â °C with measurement of the OD 595 nm every 30 min. In addition, doubling times were estimated from the OD values in the exponential part of each growth curve following a previously described method (52). These assays were performed in triplicate.

### Fluorescence Microscopy Observation.

GAS cells were grown to an OD 600 nm of 0.1 and treated with EVs (final conc. 10 µg/mL) or 20 mM Tris-HCl buffer for 2 h at 37 °C. After treatment, the cells were transferred to 12-mm diameter glass coverslips (Matsunami Glass) coated with fibronectin (Sigma) in 24-well culture plates (VIOLAMO). The attached cells were washed with PBS and fixed with 4% paraformaldehyde (PFA) in PBS for 15 min. To visualize the bacterial DNA, cells were also stained with 4’,6-diamidino-2-phenylindole (DAPI).

For immunofluorescence, cells were blocked for 1 h at room temperature with a bovine serum albumin (BSA) solution (2% BSA and 0.02% sodium azide in PBS), then probed for 2 h with anti-*S. pyogenes* group A carbohydrate antibody (Abcam,1:300) in BSA solution. After washing with PBS, the cells were labeled with anti-goat IgG conjugated to Alexa Fluor 568 (Invitrogen,1:500). Confocal fluorescence micrographs were acquired with a Zeiss LSM 900 confocal microscope with the Airyscan 2 module and Zeiss Zen 3.5 software (blue edition).

We also prepared FtsZ-mNG-expressing cells as described above with the addition of erythromycin (final conc. 1 µg/mL) at each step and observed the localization of FtsZ protein in live GAS cells.

### Time-Lapse Microscopic Observations.

Bacterial cell membranes were stained with FM4-64 (Invitrogen) at 1 μg/mL for 5 min, then the cells were washed with PBS and grown at 37 °C in THY broth containing EVs (10 µg/mL) or 20 mM Tris-buffer. Cells were then transferred to a chambered coverslip of μ-slide 4 wellPh+ (Ibidi). Fluorescence images were acquired every 10 min on a Zeiss LSM 900 confocal microscope with the Airyscan 2 module and Zeiss Zen 3.5 software (blue edition). The coverslip was incubated at 37 °C in a temperature-controlled chamber of the Tempcontrol 37-2 Digital Controllers (Zeiss).

### Electron Microscopy.

For detailed investigation of GAS cell morphology, GAS cells treated with EVs for 2 or 4 h were prepared for TEM or SEM observations (see *SI Appendix* for details). For TEM observations, the prepared ultrathin sections of cells were viewed using an H-7650 transmission electron microscope (Hitachi). The number of septa and cell length were measured manually using ImageJ software (US NIH) from TEM images of the cells in the correct focal plane. The samples prepared for SEM were observed using a JEOL JSM-7900F scanning electron microscope (JEOL).

### Live/Dead Staining Assay.

Bacterial cell viability was estimated from microscopic observations using a LIVE/DEAD BacLight Bacterial Viability Kit (Molecular Probes). This kit differentially stains live cells or damaged cells using two nucleic acid stains, SYTO9 and propidium iodide (PI). SYTO9 can label all bacterial cells whether live or dead, while PI only enters cells with damaged cell membranes. After treatment with EVs for 2 or 4 h, GAS cells were stained with SYTO9 and PI mixture (1:1) in the dark at room temperature for 15 min. The cells were observed using a Zeiss LSM 900 confocal microscope with the Airyscan 2 module and Zeiss Zen 3.5 software (blue edition). We analyzed the digital images using ImageJ software (US NIH) and calculated the cell viability from the PI-stained cell area and the SYTO9-stained cell area.

### EV Association Assay.

The interaction between EVs and GAS cells was evaluated by labeling EVs with fluorescein isothiocyanate (FITC; Sigma) following the previously described method with some modifications (53). Purified EVs were incubated with 1 mg/mL FITC in 0.1 M sodium bicarbonate (pH 9.0) for 1 h at 25 °C with shaking. After incubation, FITC-labeled EVs were washed three times with PBS to remove free FITC dye. GAS cells were incubated with FITC-labeled EVs (final 10 µg/mL) for 2 h at 30 °C, then washed twice with PBS and observed by microscopy.

### PG Labeling with Fluorescent D-Amino Acids.

To visualize PG synthesis, PG was labeled with fluorescent D-amino acids following a previously described method (54). Briefly, after 2 h treatment with EVs, GAS cells were incubated with 500 µM HCC-amino-D-alanine hydrochloride (HADA; TOCRIS) in THY broth at 37 °C for 3, 15, 30, 45, and 60 min, then washed twice with PBS. To visualize the cell membrane, cells were further immune-stained as described above and observed by fluorescence microscopy.

Images were processed using ImageJ software, and the HADA signal intensity in the cell area was quantified using MicrobeJ version 5.13I (55). The outlines of cells were detected using the “Bacteria” function, and the signal intensity of HADA was measured using the “Maxima” function. The total intensity of the HADA signal in the cell area was then calculated.

### Analysis of PG Hydrolase Activity.

The PG hydrolase activity of GAS cells was examined using a zymogram gel according to a previous study (56). Preparation of sample and gel for this analysis is detailed in *SI Appendix*. The whole cell extracts were loaded onto 10% polyacrylamide gels containing GAS cells prepared as a substrate. After electrophoresis, the gels were washed twice with distilled water at room temperature for 30 min and incubated with renaturing buffer (20 mM Tris, 50 mM NaCl, 20 mM MgCl_2_, 0.5% Triton X-100, pH 7.4) at 37 °C for 24 h. Gels were stained with staining solution (0.1% methylene blue, 0.01% KOH) for 2 h at room temperature with gentle shaking, followed by destaining with distilled water.

### Ampicillin-Induced Cell Lysis Assay.

GAS cells were grown to an OD 595 nm of 0.1 and then treated with EVs (final conc. 10 µg/mL) or 20 mM Tris-buffer for 2 h at 37 °C. After treatment, the bacterial culture was further incubated for 12 h at 37 °C in THY broth supplemented with each concentration of ampicillin (0.0001, 0.001, 0.01, 0.1, 1, and 10 mg/mL). Growth analysis was performed as described in the “Growth assay” section above.

### RNA Extraction, RNA-seq, and Bioinformatic Analysis.

Total RNA was extracted from GAS cells with or without treatment of EVs using a Quick RNA Fungal/Bacterial Microprep kit (Zymo) according to the manufacturer’s instructions. Briefly, bacterial cells were grown in THY medium with EVs or Tris-HCl and collected after 1, 2, and 4 h incubation. The cells were disrupted by lysis buffer and beads, and the total RNA in the supernatant after centrifugation was purified on the column provided in the kit. Genomic DNA was removed with DNase I (New England Biolabs) during the RNA purification. Total RNA was quantified using an ND-1000 spectrophotometer (NanoDrop) and used for qRT-PCR (see *SI Appendix* for details) and RNA-seq.

For preparing the RNA-seq library, the extracted total RNA was reassessed for yield and purity using an Agilent Bioanalyzer 2100 NanoChip (Agilent). After ribosomal RNA depletion using the Illumina Ribo-Zero Plus rRNA depletion kit, all recovered RNA was processed using the Illumina Stranded Total RNA Prep kit, following the standard protocol. Completed libraries were evaluated by Bioanalyzer analysis using a High Sensitivity DNA kit, and then sequencing was performed on the MiSeq platform using the MiSeq reagent kit v3 in paired-end mode (150 cycles, 2 × 75 bp).

The sequencing data were preprocessed using Fastp v0.36 (57) to remove the adapter and low-quality sequences, and the high-quality reads were then submitted to Salmon pipeline v1.10.3 (58) with transcript data of strain JRS4 for quantification of each gene expression level. The differential expression analysis was performed using the edgeR package v3.19 in R (59) and genes with a *P*-value below 0.01 and an absolute fold-change of more thanÂ 1.5 were considered to be differentially expressed between the two groups, EV- and Tris-HCl-treated GAS. To analyze the overrepresentation of a functional class of DEGs, we performed gene ontology (GO) functional enrichment analysis using Cytoscape plug-in ClueGO v2.5.10 (60) with a custom annotation database as a reference dataset. To create a custom annotation database, GO terms were reassigned to each gene of the JRS4 genome using FACoP v2 (61) and then manually curated for use in the ClueGO analysis.

### Hyaluronic Acid Quantification Assay.

The hyaluronic acid was quantified using a Stains-All assay according to a previously described method (62) with slight modifications. GAS cells were grown to an OD 600 nm of 0.1 and then treated with EVs (final conc. 10 µg/mL) or 20 mM Tris-buffer for 2 h at 37 °C. After EV treatment, the bacterial cells were collected from the culture medium with adjusted OD values and resuspended in 500 µL of distilled water. The hyaluronic acid was extracted by mixing with 1 mL of chloroform on a vortex mixer at room temperature for 1 h, and then the aqueous phase was collected by centrifugation. Stains-All solution (20 mg Stains-All reagent (Sigma) dissolved in 50 mL formamide, 50 mL distilled water, and 16 μL acetic acid) was added to an equal volume of aqueous phase, and the absorbance of the mixture was measured at 640 nm on a microplate reader (iMark, Bio-Rad). The amount of hyaluronic acid was determined using a standard curve generated with the use of hyaluronic acid sodium salt from GAS (Merck).

### Hemolysis Assays.

Defibrinated sheep red blood cells (RBCs; Japanbio-serum) were centrifuged at 500×*g* for 5 min, washed three times with PBS, then diluted with PBS to a final concentration of 2% (v/v) for this assay. GAS cells were grown to an OD 600 nm of 0.1 and treated with EVs (final conc. 10 µg/mL) or 20 mM Tris-buffer for 2 h at 37 °C. After treatment, the bacterial culture was centrifuged and mixed with the RBC suspension. The mixture was incubated for 2 h at 37 °C and the absorbance was measured at 570 nm using a microplate reader (iMark, Bio-Rad).

### NADase Activity Assays.

NADase activity in the culture supernatants was assessed by measuring the fluorescence intensity. The supernatants from bacterial cultures were prepared as for the “hemolysis assay” and serially diluted with PBS. The supernatant was mixed with 1 mM NAD^+^ (Nacalai Tesque) and incubated at 37 °C for 3 h. To develop the reaction, 5 M NaOH was added to the reaction mixture and further incubated at room temperature for 30 min. Then, the fluorescence intensity of the remaining NAD^+^ was detected by absorbance at 340-nm excitation/460-nm emission using a VICTOR Nivo Multimode Microplate Reader (Perkin Elmer). The activity was assessed by the NAD+ hydrolysis levels.

### Skin Infection Model.

The virulence of GAS was determined using a mouse subcutaneous infection model as described previously (63). GAS cells were grown to an OD 600 nm of 0.1 and treated with EVs (final conc. 10 µg/mL) or 20 mM Tris-buffer for 2 h at 37 °C. After treatment, the OD was normalized, and the cultures were centrifuged to collect cells. After washing three times, the cells were serially diluted with PBS, and 1 × 10^4^ CFUs were subcutaneously administered to the shaved area on the back of 6-week-old male Crlj:CD1(ICR) mice (Charles River). The lesion area was imaged daily, and mice were killed after 8 d. Images were analyzed using ImageJ processing software (US NIH) to quantify the lesion size (mm^2^). The lesion area was selected using drawing/selection tools and calculated. The mouse experiment was approved by the Animal Experiment Committee of Kyoto University, Graduate School of Medicine (approved number: 23609), and performed according to the relevant international guidelines.

## Supplementary Material

Appendix 01 (PDF)

## Data Availability

RNA sequence data have been deposited in GenBank/EMBL/DDBJ under BioProject accession no. PRJDB18572 (64). All other study data are included in the article and/or *SI Appendix*.
