# Supplementary material for: Bacterial extracellular vesicles target different bacterial species, impairing cell division and diminishing their pathogenicity
Source: Proc Natl Acad Sci U S A. 2025 Apr 29;122(18):e2416652122. doi: 10.1073/pnas.2416652122 (PMC12067206; doi:10.1073/pnas.2416652122)
Supplement: Supplementary file 1 — Appendix 01 (PDF) [file pnas.2416652122.sapp.pdf]

PNAS

Extracellular vesicles act exclusively between different bacterial species, impairing cell division and diminishing the pathogenicity

Yu Kawagishi, Kazunori Murase, Anna Grebenshchikova, Junpei Iibushi, Chang Ma, Teresia Kimeu, Atsuko Minowa-Nozawa, Takashi Nozawa, Ichiro Nakagawa

Corresponding authors:

Kazunori Murase, Ph.D.
Email: murase.kazunori.3x@kyoto-u.ac.jp

Ichiro Nakagawa, D.D.S., Ph.D.
Email: nakagawa.ichiro.7w@kyoto-u.ac.jp

This PDF file includes:

Supporting text
Figures S1 to S9
Tables S1 to S4
SI References

Fig. S1. Growth assay of various bacterial species treated with different concentrations of EVs.

Fig. S2. Cell viability assay of EV-treated GAS strain JRS4.

Fig. S3. Ampicillin-induced cell lysis assay.

Fig. S4. Zymogram analysis of cell wall hydrolase activity.

Fig. S5. Transcriptional profile of virulence-related genes in EV-treated GAS.

Fig. S6. Evaluation of the activity of virulence factors in SSI-1.

Fig. S7. Assessment of GAS pathogenicity in mouse skin infection model.

Fig. S8. Assessment of FtsZ localization and Z-ring formation in EV- and flavomycin-treated GAS.

Fig. S9. Assessment of each fraction after centrifugation of density gradient.

Table S1. Summary of DEGs showing upregulation or downregulation in EV-treated GAS.

Table S2. List of the enriched GO terms in DEGs showing the downregulation in EV-treated GAS at 4 h.

Table S3. Bacterial strains and plasmids used in this study.

Table S4. Primers used in this study.

Supporting Information Text

Methods

Construction of mutant strains

Gene deletion mutant strains were constructed by double-crossover recombination using the thermo-sensitive suicide vector pSET4S as described previously (1). Briefly, the upstream and downstream regions of the target gene were amplified from genomic DNA by PCR with the appropriate primer pair (Table S4), and the amplicon was ligated to SmaI-digested pSET4s using Gibson Assembly Mastermix (New England BioLabs). The recombinant vector containing the assembled DNA was then transformed into *S. pyogenes* (GAS) strains by electroporation, and grown on THY agar plates containing spectinomycin for 2 days at 28 °C. The colonies were replated onto fresh THY agar plates and further grown at 37 °C overnight to select single-crossover mutants. Single-crossover mutant colonies were passaged several times at 28 °C without antibiotics to remove the Spec cassette, and we finally obtained double-crossover mutants with the target gene deleted.

For construction of the JRS4 strain expressing FtsZ-mNeonGreen fusion (FtsZ-mNG) protein, the *ftsZ* gene of JRS4 and fully synthesized mNeonGreen were amplified individually by PCR with the appropriate primer pair (Table S4). These two amplicons were joined and cloned into NcoI/EcoRI-digested pAT18 using Gibson Assembly Mastermix (New England BioLabs). The recombinant vector was transformed into the wild-type strain of JRS4 by electroporation, and grown on THY agar containing erythromycin to select a clone expressing FtsZ-mNG.

Purification of isolated EVs

The isolated EVs were further purified using the OptiPrep (Sigma-Aldrich) density gradient method according to the previous report (2). The discontinuous iodixanol gradient was generated by sequentially layering 1.75 ml of 40%, 20%, 10% (wt/vol) iodixanol solutions, and 1.25 mL of 5% (wt/vol) iodixanol solutions in a PC centrifuge tube (Eppendorf). A 0.5 ml volume of EVs in PBS was overlaid on discontinuous iodixanol gradient and ultracentrifuged using an S50ST swing rotor (Hitachi) at 160,000 × g at 4°C for 16 h. Twelve fractions of each were collected from the top of the solution and subjected to Western blot analysis (Fig. S9). The fraction corresponding to EVs (fraction #9 and #10) was washed with ten times volume of PBS, and the pellet after centrifugation was resuspended in 20 mM Tris-HCl buffer for subsequent experiments.

Quantitative reverse transcription-PCR (qRT-PCR)

For qRT-PCR, total RNA was reverse transcribed using the PrimeScript II 1st strand cDNA Synthesis Kit (Takara) according to the manufacturer's instructions, and the synthesized cDNA was used as template for real-time PCR assays. Real-time PCR assays were carried out in a CFX Connect Real-Time PCR Detection System (Bio-Rad) using SsoAdvanced Universal SYBR Green Supermix (Bio-Rad), following the manufacturer's instructions. Primers for this assay were designed using Primer3 and are listed in Table S4 (3). All reactions were conducted under the following conditions: 95 °C for 20 s followed by 40 cycles of 95 °C for 10 s and 56 °C for 30 s, and then dissociation curve analysis was performed to check for non-specific amplification and primer dimer formation. The normalized expression levels of the target gene were calculated relative to the *recA* gene using the $2^{-\Delta\Delta CT}$ method (4).

Sample preparation for electron microscopy

For sample preparation for transmission electron microscopy**,** GAS cells were fixed overnight at 4 °C in 0.1 M HEPES (pH 7.4) with 2.5 % glutaraldehyde and 4 % paraformaldehyde, and washed with 0.1 M phosphate buffer (PB). After postfixation with 1 % OsO_4 in 0.1 M PB for 2 h, the cells were dehydrated with increasing concentrations of ethanol (50 %, 60 %, 70 %, 80 %, 90 %, 99 %, 100 %) for 15 min and propylene oxide for 20 min twice. The dehydrated cells were then filtered in 1:1 propylene oxide: epon for 1.5 h, 1:3 propylene oxide: epon (Luveak 812: Nacalai Tesque) for 1.5 h, and then finally polymerized with epon at 45 °C overnight and 60 °C for two nights. After cutting the embedded sample into 60–80 nm sections with an EM UC7 ultramicrotome (Leica) and staining with uranyl acetate and lead citrate.

For sample preparation for scanning electron microscopy, GAS cells were transferred to a poly-lysine-coated chamber slide (Nunc) and fixed as described above. After postfixation with 1 % OsO_4 in 0.1 M PB for 2 h, cells were dehydrated with increasing concentrations of ethanol (50 %, 60 %, 70 %, 80 %, 90 %, 99 %, 100 %) for 10–20 min, 100 % ethanol twice for 20–30 min, and t-butyl alcohol twice for 20–30 min. The cells were dried in a JFD-320 freeze dryer (JEOL) and coated with platinum using a JEC-3000FC ion sputter-coater (JEOL).

Preparation of sample and gel for zymogram analysis
After 2 h of treatment with EVs or Tris-HCl, bacterial cells were collected from 20 mL of the culture medium with an OD of 0.3 by centrifugation. The whole cell extracts were prepared by dissolving the cell pellets in SDS sample buffer and heating at 95 °C for 10 min prior to electrophoresis. For preparation of the gel containing the cell substrate, cells collected from 250 mL of culture at an OD 600 nm of 0.8 were resuspended in 1.5 M Tris-HCl (pH 8.8) and heat inactivated at 95 °C for 10 min before mixing into the resolving gel.

Fig. S1. Growth assay of various bacterial species treated with different concentrations of EVs. The growth of five different bacterial species (*S. pyogenes*, *S. epidermidis*, *B. subtilis*, *S. pneumoniae,* and *E. coli*) under each condition were measured at OD 595 nm, and the doubling times were also estimated from OD values in the exponential part of each growth curve. As for the cultivation of *S. pneumoniae*, the cells were grown in THY medium supplemented with 200 U/ml catalase under 5 % CO_2. Tris-HCl treatment was used as a control. The data represents the mean value of three biological replicates ± SEM. P-values were calculated by a two-tailed Student's t-test and denoted when the data was significant. *, $p < 0.05$; **, $p < 0.01$.

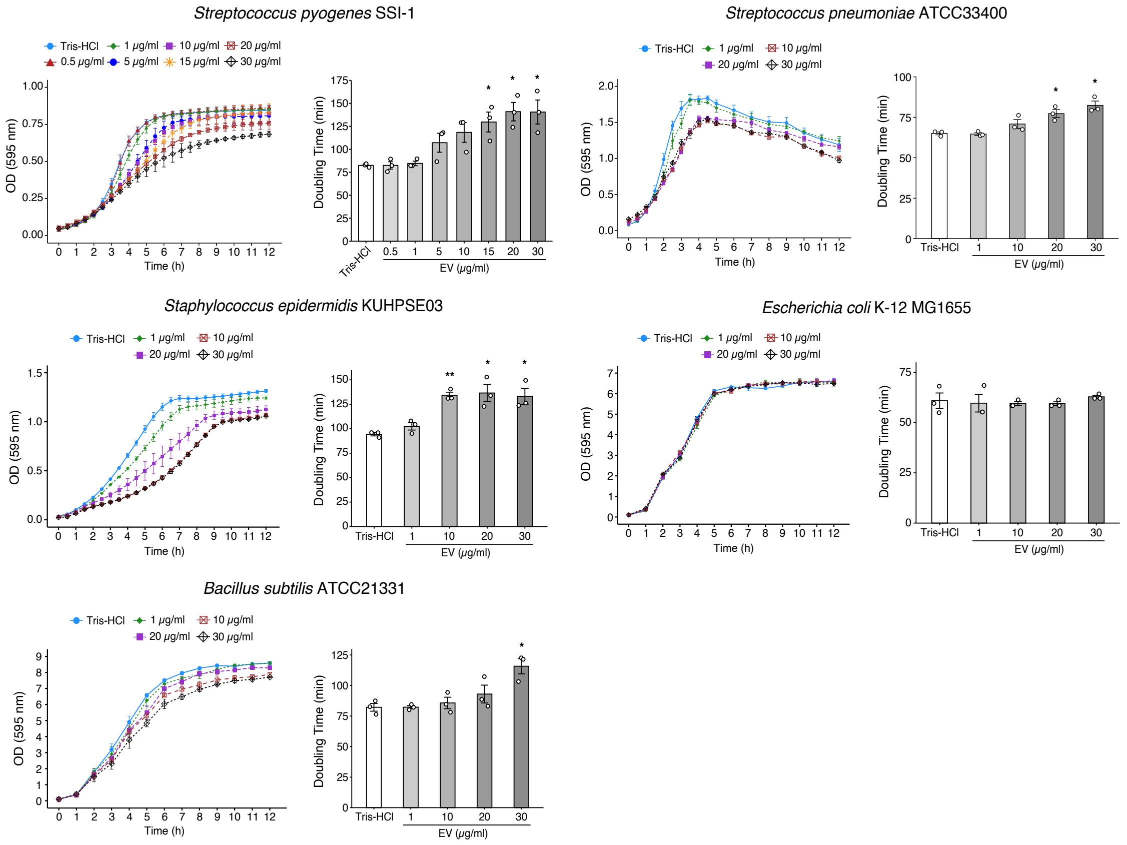

Fig. S2. Cell viability assay of EV-treated GAS strain JRS4. After treatment with EV (10 µg/mL) for 2 or 4 h, bacterial cells were stained with PI and SYTO9 and observed by confocal microscopy (Fig. 1C). The cell viability was estimated by the ratio of dead cells to total cells and represented by a bar plot with the mean value of three biological replicates ± SEM. P-values were calculated by a two-tailed Student's t-test. Cells treated with 70 % ethanol were used as a positive control (PC).

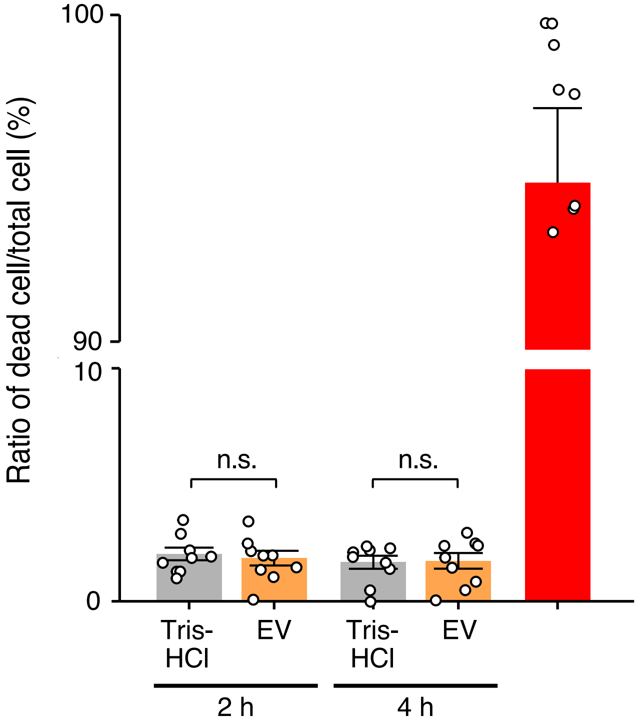

Fig. S3. Ampicillin-induced cell lysis assay. GAS cells treated with Tris-HCl or 10 μg/mL EVs for 2 h were exposed to each concentration of ampicillin (0.0001, 0.001, 0.01, 0.1, 1, and 10 mg/mL). The growth was measured at OD 595 nm and the data represent the mean value of three biological replicates ± SEM.

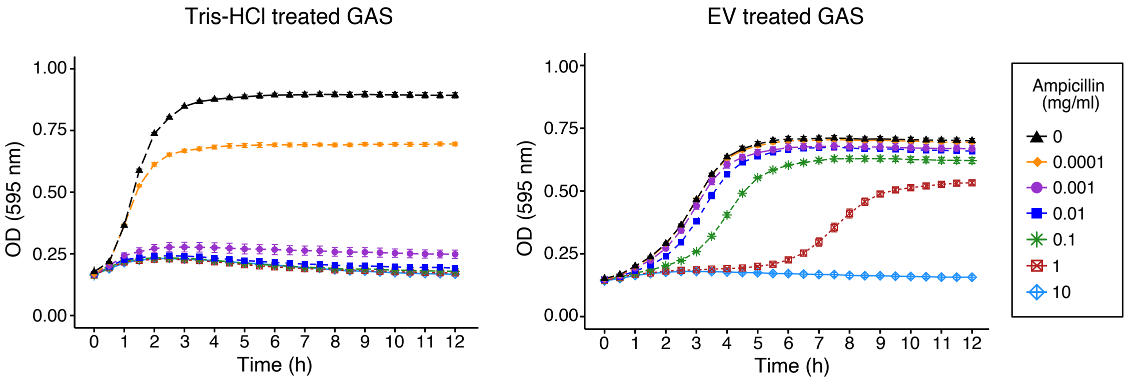

Fig. S4. Zymogram analysis of cell wall hydrolase activity. Whole cell extracts of JRS4 treated with EV or Tris-HCl for 2 h were analyzed by zymogram gel containing the cell substrate. Black arrow indicates the transparent band showing the GAS cell wall hydrolytic activity in Tris-HCl treatment, but attenuated in EV treatment. Also, no transparent bands were found in EV (10 μg or 20 μg) and *E. coli* K-12 MG1655 whole cell extract samples. M, marker.

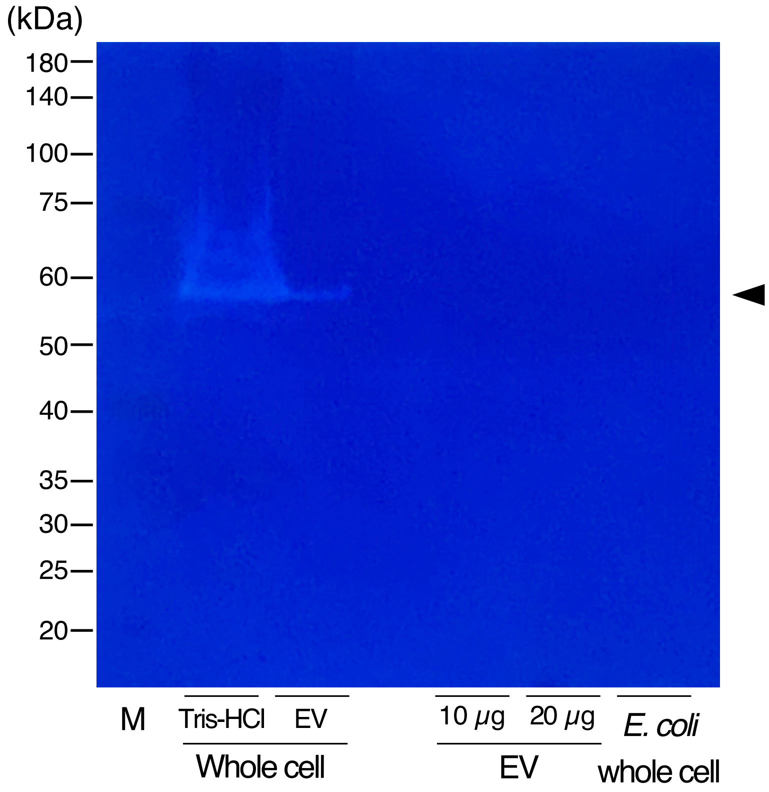

Fig. S5. Transcriptional profile of virulence-related genes in EV-treated GAS. Transcriptional expression level of four representative virulence-related genes (*nga*, *slo*, *sagA*, and *hasA*) in EV-treated JRS4 at 2h were analyzed by qRT-PCR. The normalized expression levels of each target gene were calculated relative to *recA* gene using the $2^{-\Delta\Delta}$CT method. The data represents the mean value of three biological replicates ± SEM. P-values were calculated by two-tailed Student's test.

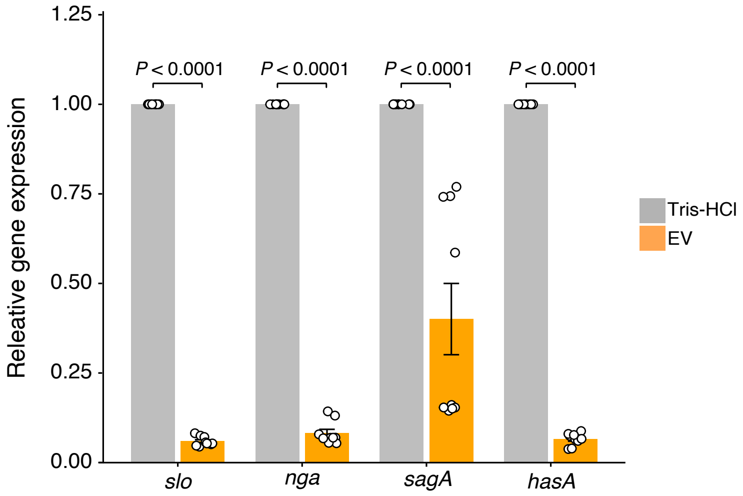

Fig. S6. Evaluation of the activity of virulence factors in SSI-1. The activities of virulence factors in SSI-1 were evaluated by NADase assay (A), hemolysis assay (B), and HA capsule production (C). Detailed methods for each assay are described in Materials and Methods. NADase activity and HA production were calculated from relative to the WT strain treated with Tris-HCl. In the hemolytic assay, the percentage of hemolysis in each sample was calculated by setting the positive control, in which RBCs were completely lysed the RBCs, at 100%. Analyzed data were shown in the bar-plots with individual data plots. The deletion mutants of genes involved in each virulence activity were used in the assay (*hasA* gene mutant for HA production, *nga* gene mutant for NADase assay, and *slo* or *sagA* gene mutant for hemolysis assay) The data represents the mean value of three biological replicates ± SEM. P-values were calculated by two-tailed Student's test.

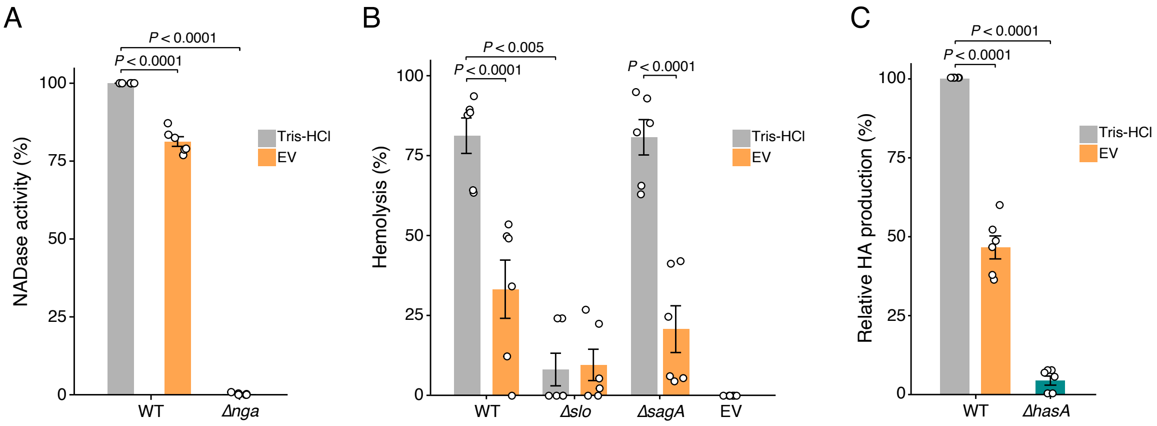

Fig. S7. Assessment of GAS pathogenicity in mouse skin infection model.

(A) Images of lesion areas in all mice at 8 dpi. In mice infected with SSI-1 treated with EV or Tris-HCl. The lesions were observed in 10 (of 14) and 11 (of 11) mice, respectively. Representative images are shown in Fig. 5D.

(B) Comparison of lesion sizes among mice with lesions. The size of lesion shown in Fig. S7A was quantified using ImageJ software. The quantified data was compared between EV or Tris-HCl treatment condition and shown in bar-plot with individual data plots. All data were obtained from the analysis with at least two biological replicates ± SEM. P-values were calculated by two-tailed Student's test.

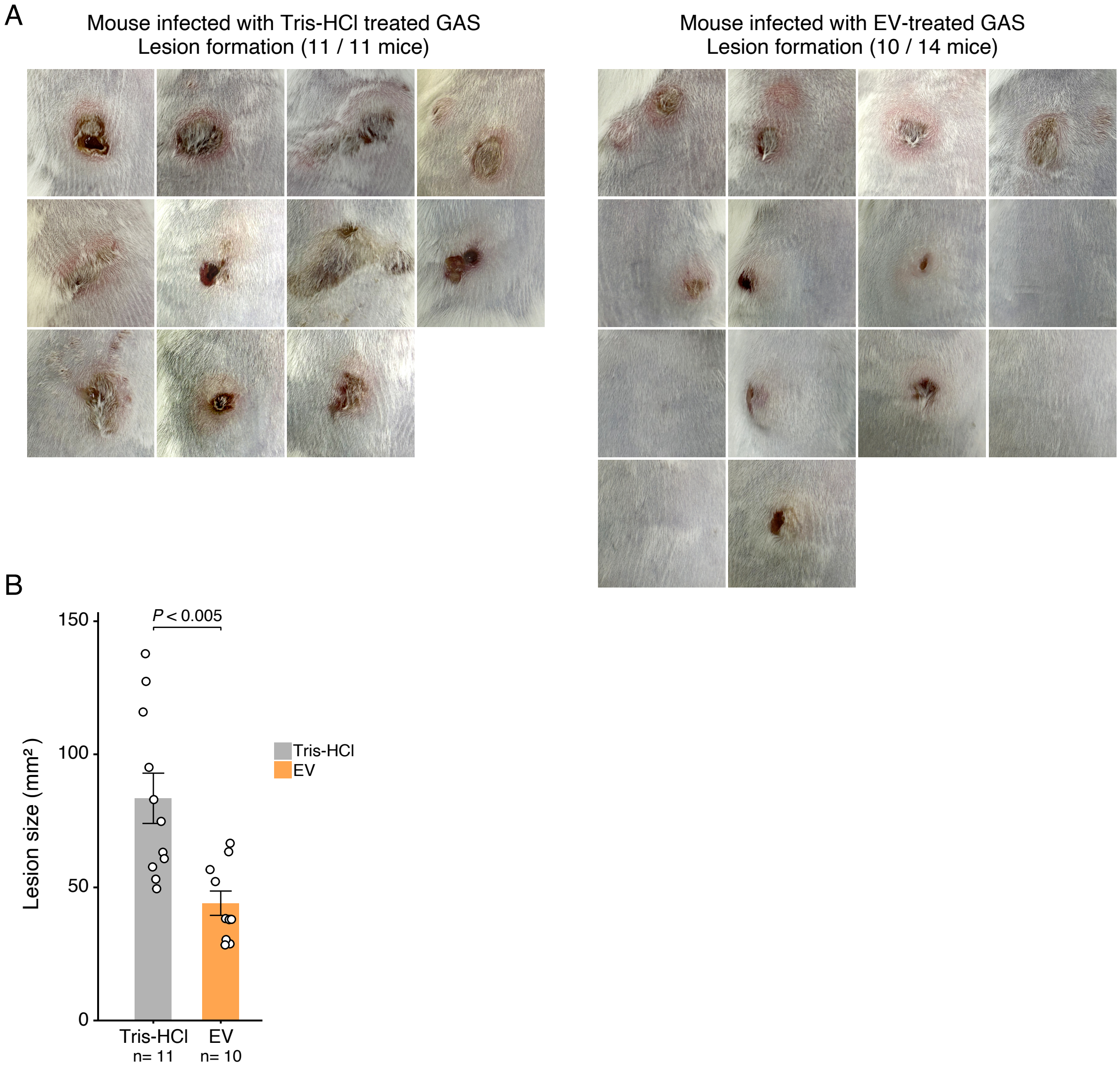

Fig. S8. Assessment of FtsZ localization and Z-ring formation in EV- and flavomycin-treated GAS.

(A) JRS4 strain expressing FtsZ-mNG (green) was treated with EVs (10 µg/mL; 500 EV particles/cell), flavomycin (0.01 µg/mL), or Tris-HCl for 2 h. Bacterial cell membranes were stained with an anti-GAC antibody (red) and observed using confocal microscopy. Cells were classified into three groups based on FtsZ localization and/or Z-ring formation: (1) single Z-ring cells—cells with a single Z-ring; (2) multiple Z-ring cells—cells containing two or more Z-rings formed in close proximity; and (3) cells with mislocalized FtsZ (characterized by diffuse or aberrant distribution) or no Z-ring formation. Scale bar, 1 µm. Representative images corresponding to the three FtsZ localization patterns (septal patterns 1, 2, and 3) are shown below for each experimental condition. These pattern images were extracted from multiple whole-cell images, including the representative whole-cell image shown above.

(B) The frequency of each type shown in panel A was analyzed relative to the total cell population. Data were obtained from three independent biological experiments, with 58 to 71 cells analyzed per condition. For quantification, chained bacterial cells were counted as single cells. The proportion of cells without detectable FtsZ fluorescence signals is not shown.

(C) The percentage of cells with multiple Z-rings relative to the total cell count (data from panel B) is presented as bar plots with individual data points. Statistical analysis was performed using the Kruskal-Wallis one-way ANOVA test, followed by an uncorrected Dunn's test for multiple comparisons.

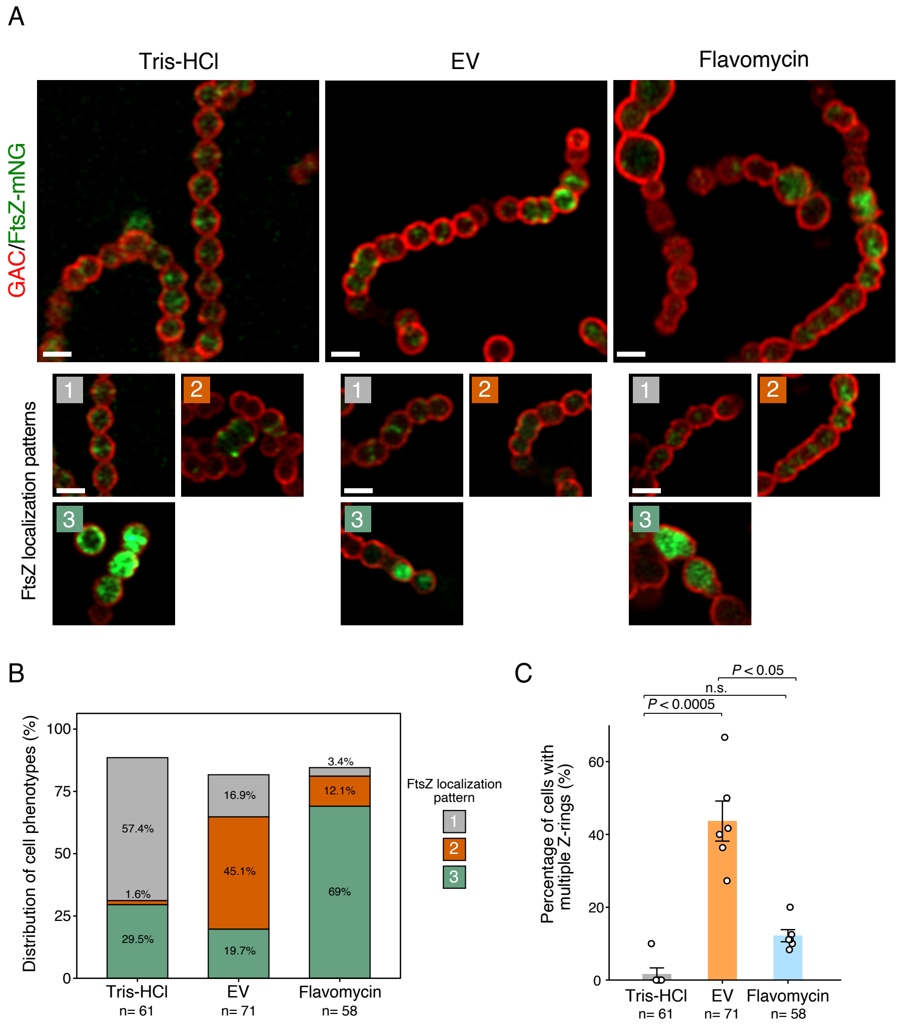

Fig. S9. Assessment of each fraction after centrifugation of density gradient. Isolated EVs were further purified by density gradient ultracentrifugation method. Twelve gradient fractions were collected and analyzed by Western blotting with anti-OmpC antibody. M, marker.

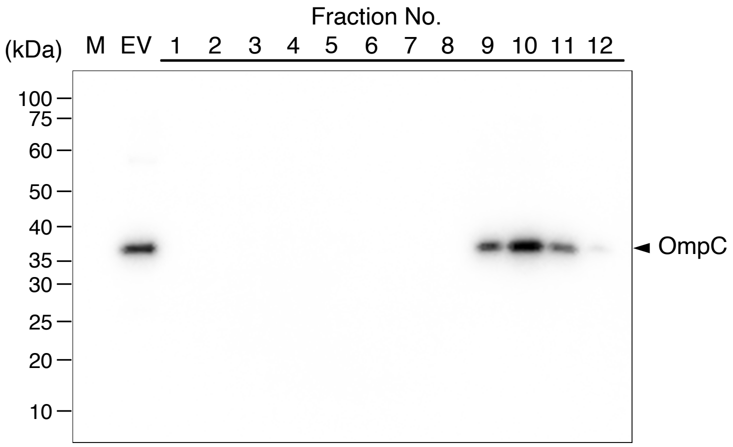

Table S1. Summary of DEGs showing upregulation or downregulation in EV-treated GAS.

	No. of DEGs*		
Under EV treatment	1h	2h	4h
Upregulation	0	11	72
Downregulation	0	15	174
Total	0	26	246

* Genes that are significantly upregulated (log^2FC >1.5, p-value < 0.01) or downregulated (log^2FC < -1.5, p-value < 0.01) in EV-treated GAS relative to untreated.

Table S2. List of the enriched GO terms in DEGs showing the downregulation in EV-treated GAS at 4 h.

GO ID	GO term	GO category*	Term p-value	Term p-value Corrected with Bonferroni step down	Group p-value	Group p-value Corrected with Bonferroni step down	GO levels	GO groups	Associated genes (%)	No. of genes
GO:0016830	carbon-carbon lyase activity	MF	0.00	0.01	0.00	0.00	[3]	Group00	39.29	11
GO:0004553	hydrolase activity, hydrolyzing O-glycosyl compounds	MF	0.00	0.00	0.00	0.00	[4]	Group01	50.00	9
GO:1901566	organonitrogen compound biosynthetic process	BP	0.00	0.00	0.00	0.00	[3]	Group02	3.86	10
GO:0006113	fermentation	BP	0.00	0.01	0.00	0.00	[5]	Group03	83.33	5
GO:0033037	polysaccharide localization	BP	0.00	0.00	0.00	0.00	[3]	Group04	100.00	5
GO:0015774	polysaccharide transport	BP	0.00	0.00	0.00	0.00	[4, 5]	Group04	100.00	5
GO:0015772	oligosaccharide transport	BP	0.00	0.01	0.00	0.00	[5]	Group04	83.33	5
GO:1901265	nucleoside phosphate binding	MF	0.00	0.04	0.00	0.00	[3]	Group05	6.17	20
GO:1901363	heterocyclic compound binding	MF	0.00	0.03	0.00	0.00	[3]	Group05	6.14	21
GO:0000166	nucleotide binding	MF	0.00	0.04	0.00	0.00	[4]	Group05	6.17	20
GO:0044282	small molecule catabolic process	BP	0.00	0.00	0.00	0.00	[3]	Group06	41.30	19
GO:0046365	monosaccharide catabolic process	BP	0.00	0.00	0.00	0.00	[4, 5]	Group06	75.00	6
GO:0170040	proteinogenic amino acid catabolic process	BP	0.00	0.03	0.00	0.00	[4, 5, 6, 7]	Group06	54.55	6
GO:0170035	L-amino acid catabolic process	BP	0.00	0.03	0.00	0.00	[5, 6, 7, 8]	Group06	54.55	6

GO:0006139	nucleobase-containing compound metabolic process	BP	0.00	0.00	0.00	0.00	[3]	Group07	5.52	23
GO:0044249	cellular biosynthetic process	BP	0.00	0.00	0.00	0.00	[3]	Group07	3.83	16
GO:0009059	macromolecule biosynthetic process	BP	0.00	0.00	0.00	0.00	[3, 4]	Group07	4.13	14
GO:0090304	nucleic acid metabolic process	BP	0.00	0.00	0.00	0.00	[3, 4]	Group07	4.20	12
GO:0010467	gene expression	BP	0.00	0.00	0.00	0.00	[4, 5]	Group07	3.64	10
GO:0003676	nucleic acid binding	MF	0.00	0.00	0.00	0.00	[3]	Group07	3.32	10
GO:0003677	DNA binding	MF	0.00	0.02	0.00	0.00	[4]	Group07	3.55	6
GO:0005975	carbohydrate metabolic process	BP	0.00	0.00	0.00	0.00	[3]	Group08	34.62	36
GO:0044282	small molecule catabolic process	BP	0.00	0.00	0.00	0.00	[3]	Group08	41.30	19
GO:0005996	monosaccharide metabolic process	BP	0.00	0.00	0.00	0.00	[3, 4]	Group08	50.00	13
GO:0016052	carbohydrate catabolic process	BP	0.00	0.00	0.00	0.00	[3, 4]	Group08	41.86	18
GO:0009311	oligosaccharide metabolic process	BP	0.00	0.00	0.00	0.00	[4]	Group08	92.31	12
GO:0009313	oligosaccharide catabolic process	BP	0.00	0.00	0.00	0.00	[4, 5]	Group08	100.00	10
GO:0019318	hexose metabolic process	BP	0.00	0.00	0.00	0.00	[4, 5]	Group08	47.62	10
GO:0046365	monosaccharide catabolic process	BP	0.00	0.00	0.00	0.00	[4, 5]	Group08	75.00	6
GO:0005984	disaccharide metabolic process	BP	0.00	0.00	0.00	0.00	[5]	Group08	92.31	12
GO:0006012	galactose metabolic process	BP	0.00	0.01	0.00	0.00	[5, 6]	Group08	83.33	5
GO:0046352	disaccharide catabolic process	BP	0.00	0.00	0.00	0.00	[5, 6]	Group08	100.00	10
GO:0006810	transport	BP	0.00	0.00	0.00	0.00	[3]	Group09	22.18	53
GO:0055085	transmembrane transport	BP	0.00	0.00	0.00	0.00	[2, 4]	Group09	24.52	51

GO:0008643	carbohydrate transport	BP	0.00	0.00	0.00	0.00	[4]	Group09	64.71	33
GO:0022857	transmembrane transporter activity	BP	0.00	0.00	0.00	0.00	[3, 5]	Group09	22.16	39
GO:0034219	carbohydrate transmembrane transport	BP	0.00	0.00	0.00	0.00	[3, 5]	Group09	63.04	29
GO:0098657	import into cell	BP	0.00	0.00	0.00	0.00	[4]	Group09	50.00	25
GO:0098739	import across plasma membrane	BP	0.00	0.00	0.00	0.00	[3, 5]	Group09	56.82	25
GO:0015144	carbohydrate transmembrane transporter activity	BP	0.00	0.00	0.00	0.00	[4, 6]	Group09	71.43	20
GO:0016310	phosphorylation	BP	0.00	0.00	0.00	0.00	[5]	Group09	29.27	24
GO:0022804	active transmembrane transporter activity	BP	0.00	0.00	0.00	0.00	[4, 6]	Group09	27.73	33
GO:0098704	carbohydrate import across plasma membrane	BP	0.00	0.00	0.00	0.00	[4, 6]	Group09	62.50	25
GO:0008982	protein-N(PI)-phosphohistidine-sugar phosphotransferase activity	BP	0.00	0.00	0.00	0.00	[5, 7]	Group09	71.43	15
GO:0009401	phosphoenolpyruvate-dependent sugar phosphotransferase system	BP	0.00	0.00	0.00	0.00	[5, 7]	Group09	61.54	24
GO:0016772	transferase activity, transferring phosphorus-containing groups	MF	0.00	0.00	0.00	0.00	[3]	Group09	23.02	32
GO:0016301	kinase activity	MF	0.00	0.00	0.00	0.00	[4]	Group09	29.89	26
GO:0016773	phosphotransferase activity, alcohol group as acceptor	MF	0.00	0.00	0.00	0.00	[4]	Group09	39.39	26

*BP, Biological Process; MF, Molecular Function

Table S3. Bacterial strains and plasmids used in this study.

Stains or plasmids	Relevant genotype*	References
Strain		
Streptococcus pyogenes		
JRS4 wild type	*Streptococcus pyogenes* str. JRS4, serotype M6	(5)
JRS4 Δ*slo*	*slo* gene deletion mutant of JRS4	(6)
JRS4 Δ*sagA*	*sagA* gene deletion mutant of JRS4	(6)
JRS4 Δ*hasA*	*hasA* gene deletion mutant of JRS4	(6)
JRS4 Δ*nga*	*nga* gene deletion mutant of JRS4	(6)
JRS4 FtsZ-mNG	JRS4 transformed with pAT18-*ftsZ*-mNeonGreen, Em^R	This study
SSI-1 wild type	*Streptococcus pyogenes* str. SSI-1, serotype M3	(7)
SSI-1 Δ*slo*	*slo* gene deletion mutant of SSI-1	(6)
SSI-1 Δ*sagA*	*sagA* gene deletion mutant of SSI-1	This study
SSI-1 Δ*hasA*	*hasA* gene deletion mutant of SSI-1	(6)
SSI-1 Δ*nga*	*nga* gene deletion mutant of SSI-1	This study
Escherichia coli		
MG1655 wild type	*Escherichia coli* K-12 str. MG1655	(8)
MG1655 GST-HlyF	*Escherichia coli* K-12 str. MG1655 transformed with pGEX6p-1_*hlyF,* Amp^R	(9)
Bacillus subtilis		
ATCC21331 wild type	*Bacillus subtilis* str. ATCC21331	(10)
Staphylococcus epidermidis		
KUHPSE03 wild type	*Staphylococcus epidermidis* str. KUHPSE03	(11)
Streptococcus pneumoniae		
ATCC33400 wild type	*Streptococcus pneumoniae* str. ATCC33400	(12)
Plasmid		
pSET4S	Shuttle vector for gene replacement; $Spec^R$	(1)
pAT18	Shuttle expression vector with *recA* promoter; Em^R	(6, 13)
pAT18-*ftsZ-mNeonGreen*	pAT18 vector expressing *ftsZ*-mNeonGreen fusion protein; Em^R	This study

*Amp^R, ampicillin resistance; Em^R, erythromycin resistance; $Spec^R$, spectinomycin resistance.

Table S4. Primers used in this study.

Primer	Oligonucleotide (5’-3') *
For construction of *nga* deletion mutant	
SSI-1_ngaKO_up_F	**tgaattcgagctcggtaccc**caatattttgcgcgtcgaaa
SSI-1_ngaKO_up_R	**ttggcaccttatacatattg**gtaaaccaccttatattatt
SSI-1_ngaKO_down_F	**aataatataaggtggtttac**caatatgtataaggtgccaa
SSI-1_ngaKO_down_R	**gtcgactctagaggatcccc**tctgactttttttcttcttt
For construction of *sagA* deletion mutant	
SSI-1_sagAKO_up_F	**tgaattcgagctcggtaccc**tttactgatgtttctagcat
SSI-1_sagAKO_up_R	**tagataaggaggtaaacctt**tctatttagcatctctatgt
SSI-1_sagAKO_down_F	**acatagagatgctaaataga**aaggtttacctccttatcta
SSI-1_sagAKO_down_R	**gtcgactctagaggatcccc**ctatcgaaatggctaaagaa
For construction of pAT18-FtsZ-mNG	
ftsZ_insert_F	**tcggtaagggcgcagcc**atggatggcattttcatt
ftsZ_insert_R	CTGCAGGTTGTTGTTacggtttttaaagaatggag
mNeonGreen_insert_F	atggtgagcaagggcgagga
mNeonGreen_insert_R	**atgaccatgattacgaattc**ttacttgtacagctcgtc
For qRT-PCR	
slo_F	accgtatcagcaaaccttcc
slo_R	cactaaaggccgcttcaaca
hasA_F	tgttagcacagacctatccgt
hasA_R	cttgagcatggcgttttcct
nga_F	tggagcaatgggtagcagtt
nga_R	ttgagccgtctaatgtgtgc
recA_F	ccttggccaatcttctcacc
recA_R	ggtcgctccgccatttaagg
sagA_F	actagtgtagctgaaacaactca
sagA_R	atttacctggcgtataacttccg

*Bold indicates overlapped sequences for Gibson assembly reaction, and the linker sequences for construction of fusion protein are shown in uppercase.

SI References

1. D. Takamatsu, M. Osaki, T. Sekizaki, Thermosensitive suicide vectors for gene replacement in *Streptococcus suis*. *Plasmid* **46**, 140-148 (2001).
2. R. Imamiya *et al.*, *Escherichia coli*-Derived Outer Membrane Vesicles Relay Inflammatory Responses to Macrophage-Derived Exosomes. *mBio* **14**, e0305122 (2023).
3. S. Rozen, H. Skaletsky, Primer3 on the WWW for general users and for biologist programmers. *Methods Mol Biol* **132**, 365-386 (2000).
4. K. J. Livak, T. D. Schmittgen, Analysis of relative gene expression data using real-time quantitative PCR and the 2(-Delta Delta C(T)) Method. *Methods* **25**, 402-408 (2001).
5. J. R. Scott, P. C. Guenthner, L. M. Malone, V. A. Fischetti, Conversion of an M- group A streptococcus to M+ by transfer of a plasmid containing an M6 gene. *J Exp Med* **164**, 1641-1651 (1986).
6. A. Roobthaisong, C. Aikawa, T. Nozawa, F. Maruyama, I. Nakagawa, YvqE and CovRS of Group A *Streptococcus* Play a Pivotal Role in Viability and Phenotypic Adaptations to Multiple Environmental Stresses. *PLoS One* **12**, e0170612 (2017).
7. I. Nakagawa *et al.*, Genome sequence of an M3 strain of *Streptococcus pyogenes* reveals a large-scale genomic rearrangement in invasive strains and new insights into phage evolution. *Genome Res* **13**, 1042-1055 (2003).
8. F. R. Blattner *et al.*, The complete genome sequence of *Escherichia coli* K-12. *Science* **277**, 1453-1462 (1997).
9. K. Murase *et al.*, HlyF Produced by Extraintestinal Pathogenic *Escherichia coli* Is a Virulence Factor That Regulates Outer Membrane Vesicle Biogenesis. *J Infect Dis* **213**, 856-865 (2016).
10. Jain DK, Collins-Thompson DL, Lee H, Trevors JT, A drop-collapsing test for screening surfactant-producing microorganisms. J Microbiol Methods 13, 271–279 (1991).
11. Y. Kawagishi *et al.*, Complete genome sequence of three *Staphylococcus epidermidis* strains. *Microbiol Resour Announc* **13**, e0017924 (2024).
12. V. B. D. Skerman, V. McGowan, P. H. A. Sneath, Approved Lists of Bacterial Names (Amended). (1989).
13. P. Trieu-Cuot, C. Carlier, C. Poyart-Salmeron, P. Courvalin, Shuttle vectors containing a multiple cloning site and a lacZ alpha gene for conjugal transfer of DNA from *Escherichia coli* to gram-positive bacteria. *Gene* **102**, 99-104 (1991).
